# Clinical characteristics and risk factors for escalation to anaphylaxis from non‐severe drug hypersensitivity reaction

**DOI:** 10.1002/clt2.70047

**Published:** 2025-04-22

**Authors:** Hyo‐In Rhyou, Sung‐Ryeol Kim, Jae‐Woo Jung, Sae‐Hoon Kim, Ji‐Hyang Lee, Hye Jung Park, Kyung‐Hee Park, Hee‐Sun Park, Eun‐Hee Chung, Gil‐Soon Choi, Sujeong Kim, Min‐Suk Yang, Jung‐Yeon Shim, Young‐Il Koh, Da‐Woon Sim, Jae‐Hyun Lee, Young‐Hee Nam, Hye‐Ryun Kang

**Affiliations:** ^1^ Department of Internal Medicine Inje University Haeundae Paik Hospital Inje University College of Medicine Busan Korea; ^2^ Department of Internal Medicine Yongin Severance Hospital Yonsei University College of Medicine Yongin Korea; ^3^ Department of Internal Medicine Chung‐Ang University Hospital Seoul Korea; ^4^ Department of Internal Medicine Seoul National University Bundang Hospital Seongnam Korea; ^5^ Drug Safety Center Seoul National University Hospital Seoul Korea; ^6^ Department of Internal Medicine Gangnam Severance Hospital Yonsei University College of Medicine Seoul Korea; ^7^ Department of Internal Medicine Institute of Allergy Yonsei University College of Medicine Severance Hospital Seoul Korea; ^8^ Department of Internal Medicine Chungnam National University Hospital Daejeon Korea; ^9^ Department of Pediatrics Chungnam National University Hospital Chungnam National University School of Medicine Daejeon Korea; ^10^ Department of Internal Medicine Kosin University College of Medicine Busan Korea; ^11^ Department of Internal Medicine School of Medicine Kyungpook National University Daegu Korea; ^12^ Department of Internal Medicine Seoul Metropolitan Government‐Seoul National University Boramae Medical Center Seoul Korea; ^13^ Department of Pediatrics Kangbuk Samsung Hospital Sungkyunkwan University School of Medicine Seoul Korea; ^14^ Division of Allergy and Clinical Immunology Department of Internal Medicine Chonnam National University Hospital Chonnam National University Medical School Gwangju Korea; ^15^ Department of Internal Medicine Dong‐A University College of Medicine Busan Korea; ^16^ Department of Internal Medicine Seoul National University College of Medicine Seoul Korea

**Keywords:** anaphylaxis, drug hypersensitivity, H2 receptor antagonists, non‐steroidal anti‐inflammatory agents, penicillins

## Abstract

**Background:**

Drug hypersensitivity reaction (DHR) poses significant challenges in clinical practice, with some patients experiencing more severe reactions upon re‐exposure. Understanding the factors contributing to escalation into more severe reactions is crucial for improving patient safety. This study aimed to investigate the clinical characteristics and risk factors associated with the progression from non‐severe DHR to anaphylaxis.

**Methods:**

A multicenter retrospective study was conducted using data from a drug‐induced anaphylaxis registry across 10 university hospitals in Korea. Clinical data, including information on culprit drugs, DHR history, and the severity of reactions, were assessed.

**Results:**

Among 494 cases of drug‐induced anaphylaxis, 417 cases (84.4%) occurred without prior DHR, while 77 cases (15.6%) had a history of non‐severe DHR. Of these, 43 cases had a previous DHR to a drug of the same class, and 34 cases involved DHR to drugs of different classes. In the group with prior DHR to a drug of the same class, anaphylaxis occurring in daily life was significantly more common compared to those reacting to a different class of drug or those with no prior DHR (48.8% vs. 23.5% or 22.5%, *p* = 0.008 and < 0.001, respectively). Non‐steroidal anti‐inflammatory drugs (NSAIDs), H2 blockers, and penicillins were identified as risk factors for anaphylaxis evolving from non‐severe DHR.

**Conclusion:**

Enhanced vigilance is required for patients with a history of non‐severe DHR to NSAIDs, H2 blockers, and penicillins as re‐exposure may lead to the progress to anaphylaxis.

## 1 INTRODUCTION

Anaphylaxis is a severe, rapidly progressive systemic reaction that can result in fatal outcomes. The incidence of anaphylaxis has been reported to be increasing, although it varies by country, population, and study design.^1^ Major causes of anaphylaxis include food, drugs, and insect venom, with drugs being the most common cause of anaphylaxis among adults.^2^ Drugs are particularly notable as the leading cause of fatal anaphylaxis,^3^ and the socioeconomic burden of drug‐induced anaphylaxis is substantial, necessitating optimized preventive strategies.^4^

Despite these concerns, drug hypersensitivity reaction (DHR), including anaphylaxis, remains unpredictable, and the incidence of drug‐induced anaphylaxis in adults continues to rise.^5^ Patients with a history of DHR are advised to avoid re‐exposure to prevent future risks.^6^ However, in practice, many DHRs recur due to re‐exposure. This often occurs when accurate DHR histories are not accessible or when effective tests are not available at the time of prescription. Contributing factors include cross‐reactivity, prescribing errors by physicians, and patient negligence. Studies from the US report about 6%–12% of medication errors occur in patients with known drug allergies.^7^

Generally, patients with DHR often experience recurrent reactions that are similar to severity to previous episodes upon re‐exposure.^8^ However, in some cases, non‐severe hypersensitivity reactions can escalate to anaphylaxis, posing a considerable risk to these patients. The factors contributing to this escalation are poorly understood. Therefore, this study aimed to investigate the clinical characteristics and risk factors associated with the progression from non‐severe DHR to anaphylaxis upon re‐exposure.

## 2 METHODS

### 2.1 Study subjects

This multicenter retrospective study was conducted across 10 university hospitals in South Korea. Cases of drug‐induced anaphylaxis by reviewing diagnoses of “anaphylaxis”, “drug allergy”, or “drug hypersensitivity” among patients diagnosed at each hospital between January 2015 and December 2021. Anaphylaxis was defined as a serious systemic hypersensitivity reaction, typically rapid in onset and potentially life‐threatening, and its assessment was based on the criteria revised by the World Allergy Organization (WHO) in 2020.^9^ Previous reactions presenting with acute hypersensitivity symptoms such as itching, urticaria, and angioedema, but not meeting the criteria for anaphylaxis, were classified as non‐severe hypersensitivity reactions. The causality of the collected drug‐induced anaphylaxis cases was assessed and categorized using the WHO‐Uppsala Monitoring Center causality assessment criteria.^10^ Cases were classified into six categories: certain, probable, possible, unlikely, conditional or unassessable by experts in drug allergy. Only cases categorized as certain or probable were included in this study.

### 2.2 Data collection and classification of culprit drugs

Demographic characteristics (age, gender, height, weight), comorbidities (allergic rhinitis, asthma, atopic dermatitis, chronic urticaria, food allergy, hypertension), and previous history of DHR were reviewed from electronic medical records. Details of the drug‐induced anaphylaxis (time of symptom onset, clinical manifestations, severity, treatment), and culprit drugs (ingredient and administration route) were also obtained from electronic medical records.

The severity of drug‐induced anaphylaxis was assessed using the Ring and Messmer grading scales: grade 1 represents generalized cutaneous signs; grade 2 indicates moderate multi‐organ involvement with cutaneous signs, hypotension, tachycardia, and bronchial hyper‐reactivity; grade 3 involves severe life‐threatening multi‐organ involvement such as collapse, tachycardia or bradycardia, arrhythmia, bronchospasm (cutaneous signs may be absent or appear only after arterial blood pressure is restored); grade 4 denotes cardiac or respiratory arrest.^11^

All identified previous and current culprit drugs were confirmed for their chemical substances, and coded according to the WHO Anatomical Therapeutic Classification (ATC) system, using the first three letters of their ATC codes.^12^

This study was approved by the institutional review boards of each participating hospital, and the requirement for informed consent was waived due to the retrospective nature of the study.

### 2.3 Statistical analysis

Statistical analyses were performed using SPSS version 22.0 (IBM SPSS Inc., Chicago, IL, USA). Continuous variables were presented as mean ± standard deviation, and categorical variables as absolute numbers and percentages. The *t‐*test was used to compare continuous variables, while the chi‐square (χ^2^) test was used for categorical variables between groups. Univariate and multivariate logistic regression analyses were employed to identify significant drug‐associated risk factors (culprit drugs and route of administration) for the escalation from non‐severe hypersensitivity reactions to anaphylaxis upon re‐exposure. A *p*‐value of <0.05 was considered statistically significant, with adjustments using the Bonferroni correction for multiple comparisons.

## 3 RESULTS

### 3.1 Characteristics of drug‐induced anaphylaxis

**FIGURE 1:**
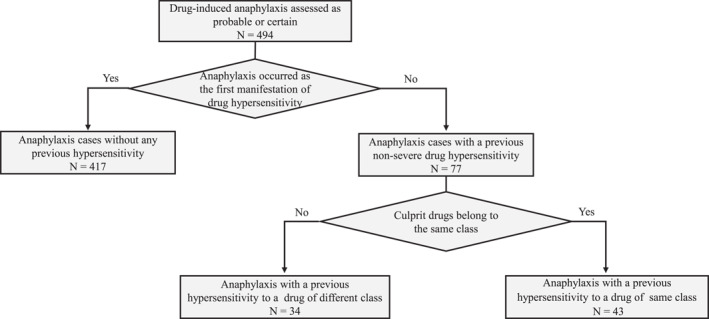
A study flow diagram describing the steps of case selection.

A total of 494 cases of drug‐induced anaphylaxis, with causality assessed as certain or probable, were collected in the study (Figure 1). Males accounted for 43.1% of the cases, and the mean age of the study subjects was 55.9 ± 22.3 years. The rates of comorbid allergic diseases were 34 (6.9%) cases of allergic rhinitis, 23 (4.7%) cases of asthma, and 6 (1.2%) cases of chronic urticaria. A history of DHR was 77 cases prior to the development of anaphylaxis, all of which were non‐severe reactions. The cases were first divided into two groups based on the presence or absence of a history of DHR (Figure 1). The former group was further divided into two subgroups based on whether the culprit drugs of the previous non‐severe hypersensitivity reactions belonged to the same class (*n* = 43) or a different class (*n* = 34) as the causative drug of anaphylaxis.

We compared the demographic and clinical characteristics of anaphylaxis among three groups: those with no prior DHR, those with prior DHR to a drug of a different class, and those with previous DHR to a drug of the same class (Table 1). There were no significant differences in sex, age, and comorbidities between the groups with a previous DHR to a drug of the same class and the other two groups. However, anaphylaxis occurring outside of the hospital setting was more common in the group with previous DHR to a drug of the same class compared to those without any previous DHR or those with DHR to a drug of a different class (48.8% vs. 22.5% or 23.5%, *p* < 0.001 and = 0.008, respectively).

**TABLE 1:** Comparison of demographic and clinical characteristics of drug‐induced anaphylaxis with and without a previous drug hypersensitivity.

Clinical variables	Anaphylaxis without any previous drug hypersensitivity *n* = 417	Anaphylaxis with a previous hypersensitivity		*p*‐value^a^	*p*‐value^b^
		To a drug of different class *n* = 34	To a drug of same class *n* = 43		
Male, *n* (%)	178 (42.7)	15 (44.1)	20 (46.5)	0.630	0.834
Age, year	60.3 ± 23.1	60.4 ± 16.6	55.5 ± 18.4	0.115	0.222
Hypertension, *n* (%)	90 (21.6)	8 (23.5)	13 (30.2)	0.381	0.446
Underlying allergic diseases, *n* (%)					
Allergic rhinitis	29 (7.0)	3 (8.8)	2 (4.7)	0.721	0.391
Asthma	20 (4.8)	1 (2.9)	2 (4.7)	0.855	0.494
Chronic urticaria	5 (1.2)	1 (2.9)	0 (0.0)	0.470	0.258
Food allergy	18 (4.3)	1 (2.9)	3 (7.0)	0.628	0.428
Instance when symptoms occurred, *n* (%)				<0.001	0.008
Hospital setting	294 (70.5)	24 (70.6)	15 (34.9)		
Outside of hospital setting	94 (22.5)	8 (23.5)	21 (48.8)		
Unknown	29 (7.0)	2 (5.9)	7 (16.3)		
Administered route of culprit drugs, *n* (%)				0.001	0.135
Intravascular	270 (64.7)	18 (52.9)	15 (34.9)		
Oral	130 (31.2)	16 (47.1)	23 (53.5)		
Subcutaneous or intramuscular	11 (2.6)	0 (0.0)	3 (7.0)		
Unknown	6 (1.4)	0 (0.0)	2 (4.7)		
Severity of anaphylaxis, *n* (%)					
Grade III or IV	343 (82.3)	23 (67.6)	32 (74.4)	0.208	0.514
Epinephrine use, *n* (%)	249 (59.7)	22 (64.7)	26 (60.5)	0.855	0.703
Systolic blood pressure, mmHg (416)	85.3 ± 34.8	99.6 ± 31.1	87.2 ± 40.2	0.764	0.210
Diastolic blood pressure, mmHg (404)	51.1 ± 22.2	63.4 ± 23.4	57.2 ± 24.7	0.143	0.345
O2 saturation, % (333)	88.1 ± 20.1	88.7 ± 14.2	93.1 ± 7.6	0.208	0.180

*Note*: Data are presented as mean ± SD or *N* (%). Significant *p*‐values adjusted at 0.025 (0.05/2) by Bonferroni method.

^a^ Values are estimated for comparison between anaphylaxis occurred without any preceding hypersensitivity group and anaphylaxis with preceding hypersensitivity to a drug belong to same class.

^b^ Values are estimated for comparison between anaphylaxis with previous hypersensitivity to drug belong to different class and anaphylaxis with preceding hypersensitivity to a drug belong to same class.

Regarding the route of administration at the time of anaphylaxis, oral administration was more common in the group with previous DHR to a drug of the same class compared to the group without any previous DHR (53.5% vs. 31.2%, *p* = 0.001). No significant differences were observed among the three groups regarding the severity of anaphylaxis, the proportion of epinephrine use, blood pressure, or oxygen saturation.

### 3.2 Culprit drugs of drug‐induced anaphylaxis

The culprit drugs responsible for drug‐induced anaphylaxis were classified according to the 3^rd^ level of ATC codes (Table 2 and Table S1). The most common culprit drug was cephalosporins (*n* = 112), followed by iodinated contrast media (ICM) (*n* = 109), non‐steroidal anti‐inflammatory drugs (NSAIDs) (*n* = 56), platins‐based chemotherapy agents (*n* = 32), H2‐receptor antagonists and proton pump inhibitors (PPIs) (*n* = 29), acetylsalicylic acid and anilides (*n* = 20), monoclonal antibodies (*n* = 17), penicillins (*n* = 13), quinolones (*n* = 13), centrally acting muscle relaxants (*n* = 13), taxanes and others (*n* = 13), and various other drugs.

**TABLE 2:** Culprit drugs of drug‐induced anaphylaxis.

Culprit agents (*n*)	Anaphylaxis without any previous drug hypersensitivity *n* (%)	Anaphylaxis with a previous hypersensitivity		*p*‐value
		To a drug of different class *n* (%)	To a drug of same class *n* (%)	
Cephalosporins (112)	96 (23.0)	5 (14.7)	11 (25.6)	0.502
Iodinated contrast media (109)	101 (24.2)	4 (11.8)	4 (9.3)	0.022
NSAIDs (56)	35 (8.4)	6 (17.6)	15 (34.9)	<0.001
Platins and others (32)	28 (6.7)	4 (11.8)	0 (0.0)	0.073
H2‐receptor antagonists and PPIs (29)	19 (4.6)	6 (17.6)	4 (9.3)	0.007
H2‐receptor antagonists	16 (3.8)	4 (11.8)	4 (9.3)	0.038
PPIs	3 (0.7)	2 (5.9)	0 (0.0)	0.046
Acetylsalicylic acid and anilides (20)	16 (3.8)	2 (5.9)	2 (4.7)	0.898
Monoclonal antibodies (17)	14 (3.4)	3 (8.8)	0 (0.0)	0.103
Penicillins (13)	8 (1.9)	2 (5.9)	3 (7.0)	0.081
Quinolones (13)	13 (3.1)	0 (0.0)	0 (0.0)	0.315
Centrally acting muscle relaxants (13)	12 (2.9)	1 (2.9)	0 (0.0)	0.624
Taxanes and others (13)	13 (3.1)	0 (0.0)	0 (0.0)	0.315
Glycopeptides (8)	7 (1.7)	0 (0.0)	1 (2.3)	0.877
Peripherally acting muscle relaxants (8)	7 (1.7)	1 (2.9)	0 (0.0)	0.665
Local anesthetics (7)	7 (1.7)	0 (0.0)	0 (0.0)	0.495
MRI contrast media (7)	7 (1.7)	0 (0.0)	0 (0.0)	0.493
Vitamin K (5)	5 (1.2)	0 (0.0)	0 (0.0)	0.999
Opioids (4)	3 (0.7)	0 (0.0)	1 (2.3)	0.483
Tetracyclines (2)	2 (0.5)	0 (0.0)	0 (0.0)	0.999
Immunosuppressants (2)	1 (0.2)	0 (0.0)	1 (2.3)	0.291
Iron preparations (2)	2 (0.5)	0 (0.0)	0 (0.0)	0.999
Acetylcysteine (2)	2 (0.5)	0 (0.0)	0 (0.0)	0.999
Others (20)	19 (4.6)	0 (0.0)	1 (2.3)	0.467

Abbreviations: MRI, magnetic resonance imaging; NSAIDs, non‐steroidal anti‐inflammatory drugs; PPIs, proton pump inhibitors.

ICM was the most common culprit drug in the group without any previous DHR, whereas NSAIDs were the most common in the other two groups with a history of DHR. Significant differences were observed in the distribution of common culprit drugs among the three groups, particularly with ICM (24.2% vs. 11.8% vs. 9.3%, *p* = 0.022), NSAIDs (8.4% vs. 17.6% vs. 34.9%, *p* < 0.001), and H2‐receptor antagonists and PPIs (4.6% vs. 17.6% vs. 9.3%, *p* = 0.007). Specifically, for anaphylaxis induced by H2‐receptor antagonists (excluding PPIs), a significant difference in distribution was still observed among the three groups (3.8% vs. 11.8% vs. 9.3%, *p* = 0.038).

**FIGURE 2:**
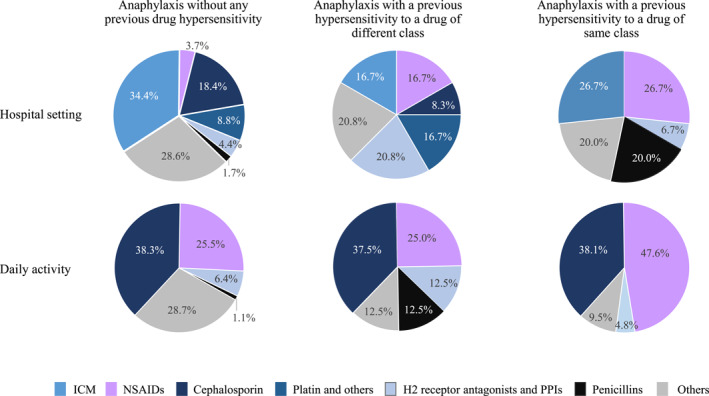
The proportion of common causative drugs by location of anaphylaxis in the study cases. ICM, iodinated contrast media; NSAIDs, non‐steroidal anti‐inflammatory drugs; PPIs, proton pump inhibitors.

The common causative drugs were further identified according to the location where anaphylaxis occurred in each of the three groups (Figure 2). The proportion of NSAIDs as the causative agent was highest in the group with a previous DHR to a drug of the same class, regardless of the location where anaphylaxis occurred. In cases that occurred within the hospital, the group without any previous DHR had the highest proportion of ICM as the causative agent compared with the other two groups.

### 3.3 Culprit drugs in anaphylaxis with a preceding drug hypersensitivity reaction

**FIGURE 3:**
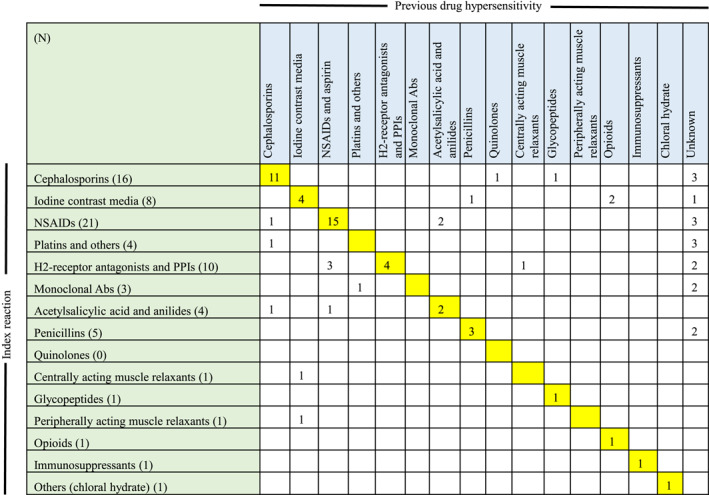
Previous culprit drugs in 77 anaphylaxis cases with a previous drug hypersensitivity reaction history. Green cells imply the culprit drugs of anaphylaxis. Blue cells imply the culprit drugs of previous hypersensitivity reaction history. Yellow cells imply the number of hypersensitivity reaction cases escalating to anaphylaxis upon re‐exposure. NSAIDs, non‐steroidal anti‐inflammatory drugs; PPIs, proton pump inhibitors.

Among 77 cases of anaphylaxis with a previous non‐severe DHR, 43 cases occurred upon re‐exposure to drugs of the same class as the previous culprit drugs. Particularly, among 21 patients with NSAID‐induced anaphylaxis, 15 (71.4%) had previously experienced non‐severe DHR to NSAIDs (Figure 3).

The active ingredients responsible for the 43 anaphylaxis cases with a preceding DHR to a drug of the same class are detailed in Table 3. In the NSAID‐induced anaphylaxis group (*n* = 15), nine cases were escalated to anaphylaxis upon re‐exposure to exactly the same ingredients: diclofenac (4), ibuprofen (3), dexibuprofen (1), naproxen (1). Three additional cases escalated to anaphylaxis from non‐severe DHR upon exposure to different NSAIDs than those initially implicated.

**TABLE 3:** List of active ingredients of 43 cases with anaphylaxis with a preceding hypersensitivity reaction to a drug of the same class.

Class of culprit agents (*n*)	Previous non‐severe reaction versus Subsequent anaphylaxis		*n*
Non‐steroidal anti‐inflammatory drugs (15)	Diclofenac	Diclofenac	4
	Ibuprofen	Ibuprofen	3
	Dexibuprofen	Dexibuprofen	1
	Naproxen	Naproxen	1
	Loxoprofen	Talniflumate	1
	Dexibuprofen, ibuprofen	Loxoprofen	1
	Aspirin, loxoprofen	Ibuprofen	1
	Unspecified NSAID	Ketorolac	2
	Unspecified NSAID	Dexibuprofen	1
Cephalosporins (11)	Cefaclor	Cefaclor	7
	Cefuroxime	Cefaclor	1
	Unspecified cephalosporin	Cefaclor	3
H2‐receptor antagonists (4)	Ranitidine	Ranitidine	3
	Ranitidine	Famotidine	1
Iodinated contrast media (4)	Iobitridol	Iopamidol	1
	Iobitridol	Iohexol	2
	Ioversol	Iohexol	1
Penicillins (3)	Amoxicillin	Amoxicillin	1
	Penicillin	Piperacillin	1
	Piperacillin	Piperacillin	1
	Unspecified ICM	Ioversol	1
Acetaminophens (2)	Acetaminophen	Acetaminophen	2
Immunosuppressants (1)	Infliximab	Infliximab	1
Glycopeptides (1)	Vancomycin	Vancomycin	1
Opioids (1)	Meperidine	Morphine	1
Chloral hydrate (1)	Chloral hydrate	Chloral hydrate	1

Abbreviations: ICM, iodinated contrast media; NSAID, non‐steroidal anti‐inflammatory drug.

In the cephalosporin group (*n* = 11), cefaclor was the unanimous culprit for anaphylaxis. The drugs responsible for previous hypersensitivity reactions were cefaclor in seven cases (63.6%), cefuroxime in one case, and unspecified cephalosporins in three cases. Among the 11 patients with cefaclor‐induced anaphylaxis, specific IgE to cefaclor was measured in four cases, all of which tested positive.

In the H2‐receptor antagonist group (*n* = 4) and the penicillin group (*n* = 3), three cases (ranitidine, *n* = 3) and two cases (amoxicillin, *n* = 1; piperacillin, *n* = 1) were triggered upon re‐exposure to the same ingredients that had caused the initial DHR. In contrast, in the ICM group, all anaphylaxis cases (*n* = 4) were triggered by the different ICM agents compared with those responsible for the initial DHR.

Among the remaining six cases, five involved re‐exposure to the same ingredient that caused the initial DHR (acetaminophen (2), infliximab (1), vancomycin (1), and chloral hydrate (1)). The last case involved anaphylaxis following morphine use in a patient with a history of previous DHR to meperidine.

### 3.4 Phenotypes of NSAID‐induced anaphylaxis with a preceding hypersensitivity reaction to NSAIDs

In cases of NSAID‐induced anaphylaxis following a preceding DHR to NSAIDs, the symptoms of both prior and subsequent reactions, along with the suggested phenotypes, are shown in Table 4. These preceding hypersensitivity reactions to NSAIDs included five instances of single NSAID‐induced urticaria/angioedema (NIUA) or anaphylaxis (SNIUAA), one case of NSAID‐exacerbated cutaneous disease (NECD), four cases of NIUA, two cases of NSAID‐exacerbated respiratory disease (NERD), and three cases where a specific phenotype could not be confirmed.

**TABLE 4:** Symptoms and phenotypes of NSAID‐induced anaphylaxis with a preceding hypersensitivity reaction to NSAIDs.

Age/sex	Previous culprit drug	Symptom of previous hypersensitivity	Culprit drug of anaphylaxis	Manifestations of anaphylaxis				Phenotype^a^
				Cutaneous	Respiratory	Gastrointestinal	Cardiovascular	
71/F	Diclofenac	Hypersensitivity NOS	Diclofenac	–	–	Vomiting	Hypotension	SNIUAA^b^
64/F	Diclofenac	Hypersensitivity NOS	Diclofenac	–	–	–	Hypotension, LOC	SNIUAA^c^
74/F	Diclofenac	Hypersensitivity NOS	Diclofenac	Urticaria, pruritus, flush	Dyspnea, chest tightness, wheezing	Nausea, vomiting	Chest pain, hypotension, LOC	SNIUAA^c^
87/M	Ibuprofen	Flush, pruritus	Ibuprofen	Pruritus	–	–	Hypotension, LOC	SNIUAA^c^
50/F	Ibuprofen	Hypersensitivity NOS	Ibuprofen	Urticaria, pruritus, flush	Dyspnea, throat tightness	–	Chest pain, hypotension, LOC	SNIUAA^c^
26/M	Unspecified NSAID	Urticaria	Ketorolac	Urticaria, flush	Dyspnea	–	–	NECD^d^
23/F	Ibuprofen	Urticaria, angioedema	Ibuprofen	Urticaria, angioedema, pruritus	Dyspnea	–	–	NIUA^d^
65/F	Dexibuprofen	Angioedema pruritus	Dexibuprofen	Angioedema, pruritus, flush	Dyspnea	Vomiting	LOC	NIUA^d^
65/M	Loxopforen	Flush, pruritus chest discomfort	Talniflumate	Angioedema, pruritus, flush	Dyspnea, chest tightness	–	LOC	NIUA^d^
44/F	Loxoprofen aspirin	Hypersensitivity NOS	Ibuprofen	Angioedema, urticaria, pruritus, flush	Dyspnea, desaturation, throat tightness, cough, wheezing, voice change	Nausea, vomiting, abdominal pain	Hypotension, chest pain	NIUA^d^
61/F	Dexibuprofen, ibuprofen	Angioedema, dyspnea, wheezing	Loxoprofen	–	Dyspnea, desaturation	Abdominal pain	–	NERD^d^
54/M	Unspecified NSAIDs	Dyspnea, wheezing	Dexibuprofen	Pruritus flush	Dyspnea, wheezing	–	Hypotension	NERD^d^
34/F	Diclofenac	Hypersensitivity NOS	Diclofenac	Flush	Chest tightness	Nausea, vomiting, abdominal pain	Palpitation, chest pain, hypotension, LOC	Unknown
54/F	Unspecified NSAID	Hypersensitivity NOS	Ketorolac	Angioedema, urticaria	Dyspnea, desaturation	–	–	Unknown
59/M	Naproxen	Hypersensitivity NOS	Naproxen	Urticaria, pruritus, flush	Dyspnea, throat tightness, cough, wheezing, voice change	–	Hypotension, palpitation	Unknown

Abbreviations: LOC, loss of consciousness; NECD, NSAIDs exacerbated cutaneous disease; NERD, NSAIDs exacerbated respiratory disease; NIUA, NSAIDs induced urticaria‐angioedema; NOS, not otherwise specified; NSAIDs, non‐steroidal anti‐inflammatory drugs; SNIUAA, single NSAID‐induced urticaria/angioedema or anaphylaxis.

^a^ In cases where NSAID phenotypes are mixed, the main phenotype is indicated.

^b^ Negative result of aspirin challenge.

^c^ History of multiple NSAID administration without any hypersensitivity reaction.

^d^ History of recurrent hypersensitivity reactions to multiple NSAIDs.

All five cases of SNIUAA presented with hypotension and loss of consciousness during the anaphylactic episode. One case was confirmed as SNIUAA through a negative result of the aspirin provocation test and the others were diagnosed as SNIUAA based on a history of uneventful administration of multiple NSAIDs other than the culprit one. Among the four cases of NIUA, three had confirmed cutaneous symptoms (rash, pruritus, urticaria, and angioedema) during the preceding hypersensitivity reactions. All NIUA cases escalated to anaphylaxis, presenting with both cutaneous and respiratory symptoms, and one case exhibited cardiovascular collapse. These patients had a history of recurrent cutaneous symptoms due to multiple NSAIDs. The two cases of NERD had histories of recurrent wheezing and asthma exacerbation following NSAID exposure, with one case also presenting with cardiovascular collapse during an anaphylactic event. A single case of NECD escalated to anaphylaxis, presenting with both cutaneous and respiratory symptoms, following a history of recurrent urticaria.

### 3.5 Drug‐associated factors for escalation of non‐severe hypersensitivity reaction to anaphylaxis upon re‐exposure

Drug‐associated risk factors for the escalation of non‐severe DHR to anaphylaxis upon re‐exposure were investigated using univariate and multivariate logistic regression analyses (Table 5). In the univariate analysis, NSAIDs (odds ratio (OR) = 5.357, 95% confidence interval (CI) = 2.648–10.837, *p* < 0.001), and the oral route of administration (OR = 2.331, 95% CI = 1.241–4.379, *p* = 0.009) were significantly associated with an increased risk of progression to anaphylaxis upon re‐exposure. Conversely, ICM (OR = 0.338, 95% CI = 0.118–0.968, *p* = 0.043) and the intravenous route of administration (OR = 0.413, 95% CI = 0.252–0.676, *p* < 0.001) were associated with a lower risk of escalation to anaphylaxis.

**TABLE 5:** Drug associated risk factors for escalation of hypersensitivity reaction to anaphylaxis upon re‐exposure.

Risk factors	Univariate analysis			Multivariate analysis		
	OR	95% CI	*p*‐value	OR	95% CI	*p*‐value
Culprit agents						
NSAIDs	5.357	2.648–10.837	<0.001	4.742	2.117–10.624	<0.001
H2 receptor antagonists	2.210	0.719–6.791	0.166	3.390	1.024–11.217	0.046
Penicillins	3.307	0.875–12.508	0.078	5.028	1.242–20.349	0.024
Iodinated contrast media	0.338	0.118–0.968	0.043	0.900	0.271–2.994	0.864
Route of administration						
Intravascular route	0.306	0.159–0.590	<0.001	0.221	0.063–0.771	0.018
Oral route	2.331	1.241–4.379	0.009	0.405	0.126–1.303	0.129

Abbreviations: CI, confidential interval; NSAIDs, non‐steroidal anti‐inflammatory drugs; OR, odds ratio.

In the multivariate analysis, NSAIDs (OR = 4.742, 95% CI = 2.117–10.624, *p* < 0.001), H2‐receptor antagonists (OR = 3.390, 95% CI = 1.024–11.217, *p* = 0.046), and penicillins (OR = 5.028, 95% CI = 1.242–20.349, *p* = 0.024) were identified as significant risk factors for escalation to anaphylaxis upon re‐exposure. In contrast to the univariate analysis, the oral route of drug administration did not emerge as a significant risk factor in the multiple analysis, while the intravascular route continued to be a significant factor associated with a reduced risk of escalation to anaphylaxis (OR = 0.221, 95% CI = 0.063–0.771, *p* = 0.018).

## 4 DISCUSSION

This study examined the clinical characteristics and culprit drugs associated with drug‐induced anaphylaxis, specifically in cases where non‐severe hypersensitivity reactions escalated to anaphylaxis upon re‐exposure.

Our findings reveal that escalation from a non‐severe reaction to anaphylaxis upon re‐exposure occurred more commonly in daily life than within hospital settings. This suggests that healthcare professionals may generally exercise caution when prescribing and administering drugs to patients with a history of DHR. In the present study, the intravascular route was associated with a lower risk of escalation to anaphylaxis from non‐severe hypersensitivity reactions, which may be attributed to the cautious prescription of intravascular drugs by physicians in hospital settings. Although parenteral administration is known to be more sensitizing than the oral route,^13^ the impact of parenteral administration on escalation from a non‐severe hypersensitivity reaction to anaphylaxis remains unclear.

Nevertheless, approximately one‐third of cases where hypersensitivity reaction progressed to anaphylaxis due to re‐exposure to the same drug occurred within a hospital. The causative agents involved in repeated exposures within hospitals included NSAIDs, ICM, penicillins, vancomycin, infliximab, ranitidine and morphine. Physicians may sometimes choose to re‐administer these drugs or drugs from the same class when the previous histories are ambiguous, symptoms were not severe, or the drug is deemed essential for treatment.^14^, ^15^ However, this practice carries the risk of hypersensitivity reactions escalating to anaphylaxis. Therefore, it underscores the need for continued vigilance and caution in managing patients with a prior history of DHR. Additionally, the distribution of culprit drugs varied depending on the location of anaphylaxis occurrence, necessitating the development of tailored drug monitoring strategies.

Repeated allergen exposure can boost hypersensitivity reactions by enhancing allergen‐specific T cell activation and increasing IgE production. However, the precise mechanisms that lead to escalated severity are still not well understood.^16^

In this study, we observed significant variations in the distribution of common culprit drugs, particularly NSAIDs, H2‐receptor antagonists, penicillins, and ICMs, across the three groups: anaphylaxis without previous DHR, anaphylaxis with previous non‐severe hypersensitivity reaction to a drug of a different class, and anaphylaxis with previous non‐severe hypersensitivity reaction to a drug of the same class. Although this study did not determine the incidence of anaphylaxis relative to the total number of drug exposures nor did it provide recurrence rates, the varying distribution of culprit drugs based on prior hypersensitivity reactions suggests that the escalation of non‐severe hypersensitivity reaction to anaphylaxis upon re‐exposure may be related to specific drugs. Especially, NSAIDs, H2‐receptor antagonists, and penicillins appear to carry a higher risk of escalating from non‐severe reaction to anaphylaxis upon re‐exposure. Therefore, patients with a history of DHR to these drugs require heightened vigilance on re‐exposure.

NSAIDs emerged as the most common culprit drug in both groups with previous DHR. Notably, the proportion of NSAIDs as culprit drugs was highest in anaphylaxis cases with preceding DHR to a drug of the same class. Most hypersensitivity reactions to NSAIDs are known to be related to the inhibition of cyclooxygenase‐1, resulting in non‐selective reactions that are further classified into NERD, NECD, and NIUA.^17^ On the other hand, selective NSAID‐induced hypersensitivity reactions, which involve immunologic mechanisms, are divided into IgE‐mediated SNIUAA and non‐IgE‐mediated delayed hypersensitivity reactions.^17^ The incidence of each phenotype of NSAID hypersensitivity is unknown, and the prevalence of non‐selective NSAID hypersensitivity varies considerably across different regions. In Southeast Asia, the most prevalent NSAID hypersensitivity is NIUA, occurring in approximately 64% of cases, while NECD is identified in around 10%.^18^ In contrast, NERD is a relatively uncommon phenotype, accounting for only a small proportion of cases. In East Asia, including Korea, NERD shows a higher prevalence compared with Southeast Asia, accounting for approximately 6% of all asthma patients.^18^, ^19^ However, there is a paucity of data regarding the prevalence of SNIUAA, a rare phenotype of NSAID hypersensitivity. In this study, various phenotypes of NSAID‐induced hypersensitivity were observed in cases that escalated to anaphylaxis upon re‐exposure; however, SNIUAA was the most common phenotype. This highlights the importance of a precise confirmatory diagnostic procedure and the necessity of completely avoiding re‐exposure in patients with SNIUAA, even if previous reactions were not severe. NIUA, NECD, and NERD also appeared in the cases that escalated to anaphylaxis, underscoring the need for caution regarding the potential for escalation to anaphylaxis upon re‐exposure to NSAIDs.

In the cases where non‐severe hypersensitivity reactions escalated to anaphylaxis upon re‐exposure, most cases were triggered by NSAIDs, followed by cephalosporins, H2‐receptor antagonists, ICMs, penicillins, and acetaminophen. Specifically, in cephalosporin‐induced cases, all instances of escalation to anaphylaxis upon re‐exposure were caused by cefaclor. Specific IgE testing revealed that all cases tested positive for cefaclor‐specific IgE, indicating an IgE‐mediated response. Various reactions, including IgE‐medicated responses, can trigger cephalosporins‐induced hypersensitivity,^20^ and the escalation to anaphylaxis upon re‐exposure may also be related to IgE‐mediated reactions. IgE‐mediated reactions are also known to be common in DHR induced by H2‐receptor antagonists^21^ and penicillins.^22^ Some drugs, such as infliximab and vancomycin, can induce both infusion‐related reaction and immunologic hypersensitivity reactions, and distinguishing between these two types of responses can often be challenging for physicians.^23^, ^24^

ICM was the most common culprit drug in cases of anaphylaxis without any prior history of DHR. A retrospective study evaluating risk factors for ICM‐induced anaphylaxis among 76,194 ICM administrations found that about 70% of cases occurred in patients without any prior adverse reactions to previous ICM use.^25^ These findings suggest that the risk of ICM‐induced anaphylaxis should always be emphasized to all patients, regardless of previous history of DHR to ICM.

Additionally, most cases of escalation to anaphylaxis upon re‐exposure to ICM involved different prior and subsequent culprit ICM, as it has been recommended to switch the culprit ICM to another ICM when patients with a history of ICM‐induced hypersensitivity reaction undergo a CT scan. The N‐(2,3‐dihydroxypropyl) carbamoyl side chain may have been implicated in cross‐reactivity in ICM‐induced hypersensitivity.^26^, ^27^ However, in the present study, escalation to anaphylaxis occurred despite changing the culprit ICMs, with three of four the cases not sharing a common side chain between the previous and current ICMs. Therefore, physicians should inform patients with a history of non‐severe ICM hypersensitivity reaction about the potential risk of anaphylaxis, even if a different ICM that does not share a common side chain is used.

There are some limitations to this study. First, as a retrospective study, there may be cases where the identification of the culprit drug is unclear. However, we believe this limitation is mitigated by including only cases where causality was assessed as certain or probable. In addition, even in cases where the symptoms of previous reactions to NSAIDs were not documented in detail, NSAID hypersensitivity reactions were considered highly likely based on consultations with allergy specialists. However, the phenotypes of these reactions were predominantly classified using patient history, underscoring a limitation in confirming or validating the exact underlying mechanisms. Second, diagnostic tests, including drug skin test, serum drug specific IgE measurement, and drug provocation tests, were not universally conducted due to the lack of standardization and the potential risk of inducing severe reactions. This highlights the need for developing safe and more reliable diagnostic methods for DHR. Therefore, the development of more convenient and safe tests for DHR is required. One of the challenges in collecting epidemiological data on drug re‐exposure is the difficulty in tracking these occurrences, particularly when they happen outside of hospital settings.^7^ Therefore, heightened vigilance is warranted for patients with a history of DHR to ensure accurate record‐keeping and safety of patients.

In summary, the type of culprit drug may be related to the risk of escalation to anaphylaxis upon re‐exposure, with NSAIDs, H2‐receptor antagonists, and penicillins presenting a higher risk. Particularly, prior DHR to these drugs necessitates extensive pharmacovigilance during re‐exposure to prevent anaphylaxis.

## AUTHOR CONTRIBUTIONS

**Hyo‐In Rhyou**: Writing—original draft; writing—review and editing; visualization; formal analysis; validation; project administration. **Sung‐Ryeol Kim**: Data curation; investigation; writing—review and editing; software; methodology. **Jae‐Woo Jung**: Data curation; resources. **Sae‐Hoon Kim**: Data curation; resources. **Ji‐Hyang Lee**: Data curation; resources. **Hye Jung Park**: Data curation; resources. **Kyung‐Hee Park**: Data curation; resources. **Hee‐Sun Park**: Data curation; resources. **Eun‐Hee Chung**: Data curation; resources. **Gil‐Soon Choi**: Data curation; resources. **Sujeong Kim**: Data curation; resources. **Min‐Suk Yang**: Data curation; resources. **Jung‐Yeon Shim**: Data curation; resources. **Young‐Il Koh**: Data curation; resources. **Da‐Woon Sim**: Data curation; resources. **Jae‐Hyun Lee**: Data curation; resources; supervision. **Young‐Hee Nam**: Investigation; validation; writing—review and editing; data curation; resources; supervision. **Hye‐Ryun Kang**: Conceptualization; methodology; software; data curation; investigation; supervision; resources; writing—review and editing; visualization; funding acquisition; validation.

## CONFLICT OF INTEREST STATEMENT

The authors declare no conflicts of interest.

## Supporting information

Table S1

## Data Availability

Data sharing is not applicable to this article as no new data were created or analyzed in this study.
