# Supplementary material for: Clinical characteristics and risk factors for escalation to anaphylaxis from non‐severe drug hypersensitivity reaction
Source: Clin Transl Allergy. 2025 Apr 22;15(4):e70047. doi: 10.1002/clt2.70047 (PMC12014396; doi:10.1002/clt2.70047)
Supplement: Supplementary file 1 — Table S1 [file CLT2-15-e70047-s001.docx]

**SUPPLEMENTARY TABLES**

**Table S1.** Culprit drugs of cases with drug-induced anaphylaxis

| ATC code | Culprit agents (n) | Detailed substances |
| --- | --- | --- |
| J01D | Cephalosporins (112) | Cefaclor (58), ceftriaxone (26), cefazedone (5), cefazolin (5), cefotaxime (3), ceftezole (3), cefepime (2), cefotetan (2), cefminox (2), cefbuperazone (1), cefpiramide (1), ceftazidime (1), cephradine (1), flomoxef (1), unspecified cephalosporins (1) |
| V08A | Iodinated contrast media (109) | Iopamidol (45), iohexol (34), iobitridol (8), iomeprol (6), ioversol (6), iopromide (4), iodixanol (3), unspecified ICM (3) |
| M01A | NSAIDs (56) | Ibuprofen (12), diclofenac (10), dexibuprofen (9), ketorolac (8), aceclofenac (5), naproxen (5), celecoxib (4), loxoprofen (2), talniflumate (1) |
| L01X | Platins and others (32) | Oxaliplatin (13), carboplatin (11), cisplatin (5), pegaspargase (3) |
| A02B | H2-receptor antagonists and PPIs (29) | Ranitidine (22), famotidine (2), esomeprazole (2), lansoprazole (2), rabeprazole (1) |
| N02B | Acetylsalicylic acid and anilides (20) | Acetaminophen (16), aspirin (3), nefopam (1) |
| L01F | Monoclonal antibodies (17) | Cetuximab (10), ramucirumab (2), rituximab (5) |
| J01C | Penicillins (13) | Amoxicillin (9), piperacillin (4) |
| J01M | Quinolones (13) | Moxifloxacin (6), ciprofloxacin (4), levofloxacin (2), ofloxacin (1) |
| M03B | Centrally acting muscle relaxants (13) | Eperisone (13) |
| L01C | Taxanes and others (13) | Paclitaxel (5), docetaxel (4), irinotecan (4) |
| J01X | Glycopeptides (8) | Vancomycin (4), teicoplanin (4) |
| M03A | Peripherally acting muscle relaxants (8) | Rocuronium (8) |
| N01B | Local anesthetics (7) | Lidocaine (4), mepivacaine (1), articaine (1), procaine (1) |
| V08C | MRI contrast media (7) | Gadobutrol (3), gadoterate (2), gadoxetate (1), gadoteridol (1) |
| N02A | Opioids (4) | Tramadol (3), morphine (1) |
| L04A | Immunosuppressants (2) | Infliximab (1), tocilizumab (1) |
| B02B | Vitamin K (5) | Vitamin K (5) |
| J01A | Tetracyclines (2) | Minocycline (2) |
| B03A | Iron preparations (2) | Ferric hydroxide sucrose complex (1), iron isomoltoside complex (1) |
| R05C | Acetylcysteine (2) | Acetylcysteine (2) |
|  | Others (1 each) | Bovine thrombin, cetrizine, cerebrolysin, chloral hydrate, clopidogrel, dioctahedral smectitie, fluorouracil, fluorescein sodium, HDM-immunotherapy, hyaluronidase, herbal medicine, metoclopramide, nafamostat mesilate, pancreatin enteric coated microtablet, praziquantel, protamine sulfate, protein supplement, remifentanil, scopolamine butylbromide, sugammadex |

HDM, house dust mite; NSAIDs, non-steroidal anti-inflammatory drugs; MRI, Magnetic Resonance Imaging; PPIs, proton pump inhibitors.
